# Osteopathic Manipulative Treatment in Tension Headaches

**DOI:** 10.7759/cureus.12040

**Published:** 2020-12-12

**Authors:** Justin Chin, Wenqi Qiu, Christine M Lomiguen, Mikhail Volokitin

**Affiliations:** 1 Medical Education, Lake Erie College of Osteopathic Medicine, Erie, USA; 2 Family Medicine, LifeLong Medical Care, Richmond, USA; 3 Epidemiology and Biostatistics, State University of New York (SUNY) Downstate Health Sciences University, New York, USA; 4 Pathology, Lake Erie College of Osteopathic Medicine, Erie, USA; 5 Osteopathic Manipulative Medicine, Touro College of Osteopathic Medicine, New York, USA

**Keywords:** tension headache, headache, pain control, tension, nsaid, osteopathic manipulative medicine, omm, suboccipital, muscle energy, osteopathic care

## Abstract

Tension-type headaches, associated with young age, poor health, sleep disturbances, anxiety, stress, and poor posture, account for 90% of all headaches diagnosed by healthcare professionals. Diagnosis and treatment of the various headache subtypes are often aimed at determining the underlying cause but commonly involve over-the-counter pain medication. Because recurrence is common in tension-type headaches, with a subsequent refractory response to over-the-counter medications, adjunctive and alternative treatment modalities should be further studied. Here we present a case of tension headache initially non-responsive to pain medication but resolved with osteopathic manipulative treatment and lifestyle modifications. Osteopathic considerations and literature are also reviewed in the broader context of headache management.

## Introduction

Headaches are among the most common reasons for seeking medical care in emergency rooms and primary care offices alike [1]. Greater than 75% of people have a headache at some point in their life, with episodes typically lasting anywhere between several hours to days [2]. Headaches are generally differentiated into primary and secondary causes, with over 200 different subtypes and classifications [3]. It is important to note that the brain does not have pain receptors. However, the surrounding head and neck structures do, and pain can refer there to cause headaches [4]. Diagnosis and treatment of headaches are often aimed at determining the underlying cause, but commonly involve over-the-counter (OTC) pain medication.

In addition to pharmaceutical intervention, alternative management options can include acupuncture, physical therapy, and osteopathic manipulative medicine (OMM). OMM and its subsequent use in treatment started in the late 19th century under the auspices of Dr Andrew Taylor Still [5]. Guided by the tenets of osteopathic medicine, osteopathic physicians, also known as Doctors of Osteopathic Medicine (DO), are trained to promote the body’s natural tendency toward self-healing, health, and the interrelationship between structure and function. The bodily disease can manifest as restrictions of the fascia, joints, and muscles, in which the role of the DO is to remove these dysfunctions with osteopathic manipulative treatment (OMT), to allow for the body to return to homeostasis [6,7]. Despite its well-documented efficacy in various acute and chronic pain complaints, limited research exists about OMT and headache management [8-10].

Here we present a case of tension headache initially non-responsive to pain medication but resolved with OMT and lifestyle modifications. Osteopathic considerations and literature are also reviewed in the broader context of headache management.

## Case presentation

A 28-year-old Asian male presented to the osteopathic medicine clinic with a three-day history of steadily worsening headaches. The pain initially started at the suboccipital region and subsequently radiated to above the superior nuchal line and encircled the entire head. The patient described the pain as deep and achy, rated its severity as five (5) out of ten (10) on the Faces pain scale, and reported minimal improvement with the use of over-the-counter ibuprofen, acetaminophen, and topical capsaicin cream. Past medical, surgical, family, and social history were non-contributory. The patient denied any previous trauma, diplopia, vertigo, photophobia, sinus pressure, visual aura, or history of migraines. He, however, noted that he was under a large amount of stress due to upcoming graduate school examinations. The patient stated he typically spent greater than eight hours at a time reviewing material on his computer in the last week.

**Figure 1:**
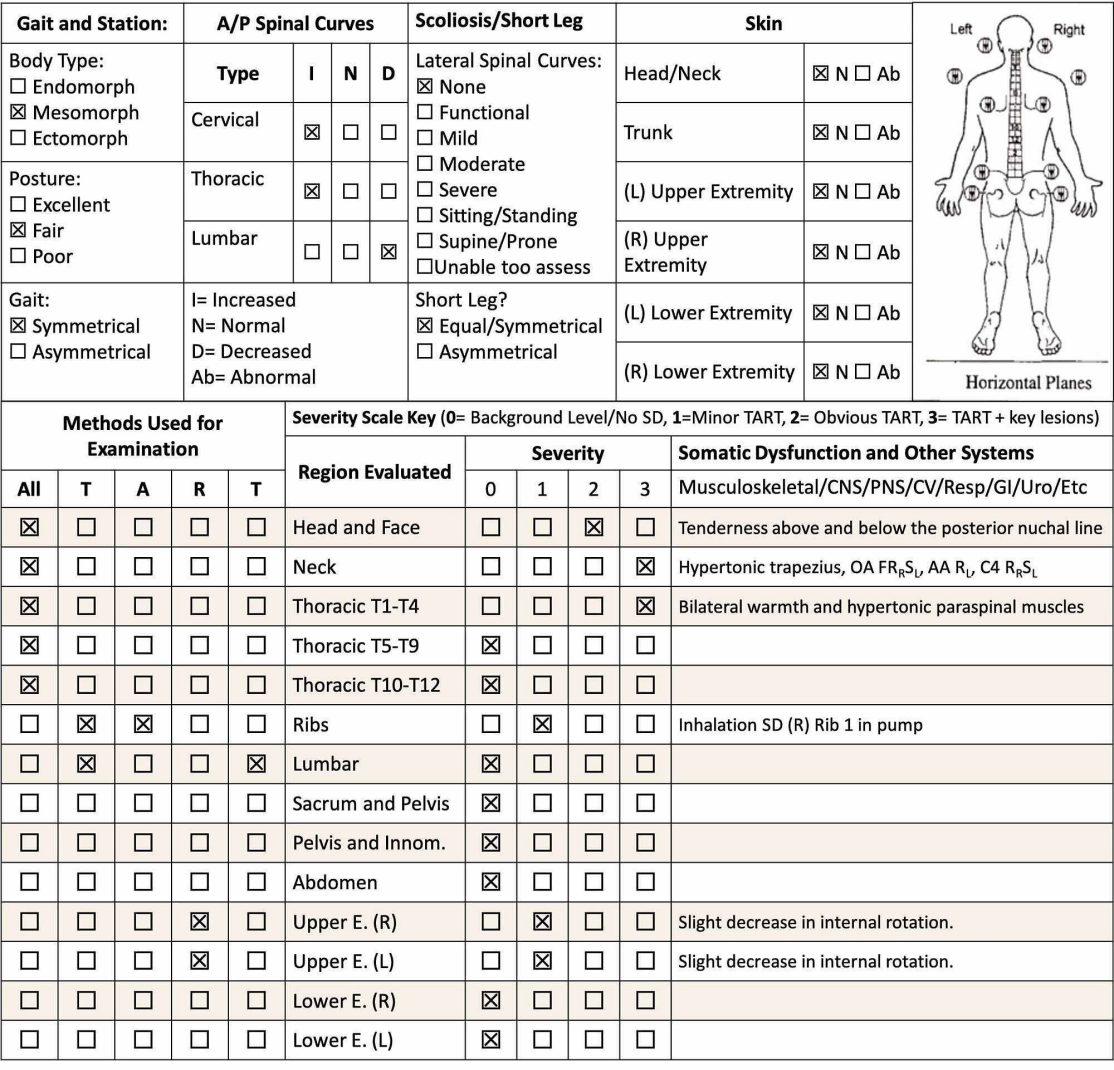
Complete osteopathic structural exam Adapted from the Journal of the American Osteopathic Association, with patient findings [6,7]. TART: tenderness, asymmetry, restricted motion, tissue texture changes.

Vitals were within normal limits. Physical exam revealed normal neurological and musculoskeletal findings, without any asymmetry in deep tendon reflexes, sensation, or strength. Full range of motion was observed in active and passive movement in all directions for the head, neck, and extremities. On osteopathic physical exam, palpation elicited tenderness of the suboccipital groove and detected hypertonicity of the bilateral upper trapezius muscles. The following somatic dysfunctions were noted in conjunction with the aforementioned physical exam: flexed atlantooccipital joint with right rotation and left side bending (OA FRRSL), atlantoaxial rotation to the left (AA RL), and various thoracic vertebra problems (Figure 1).

**Figure 2:**
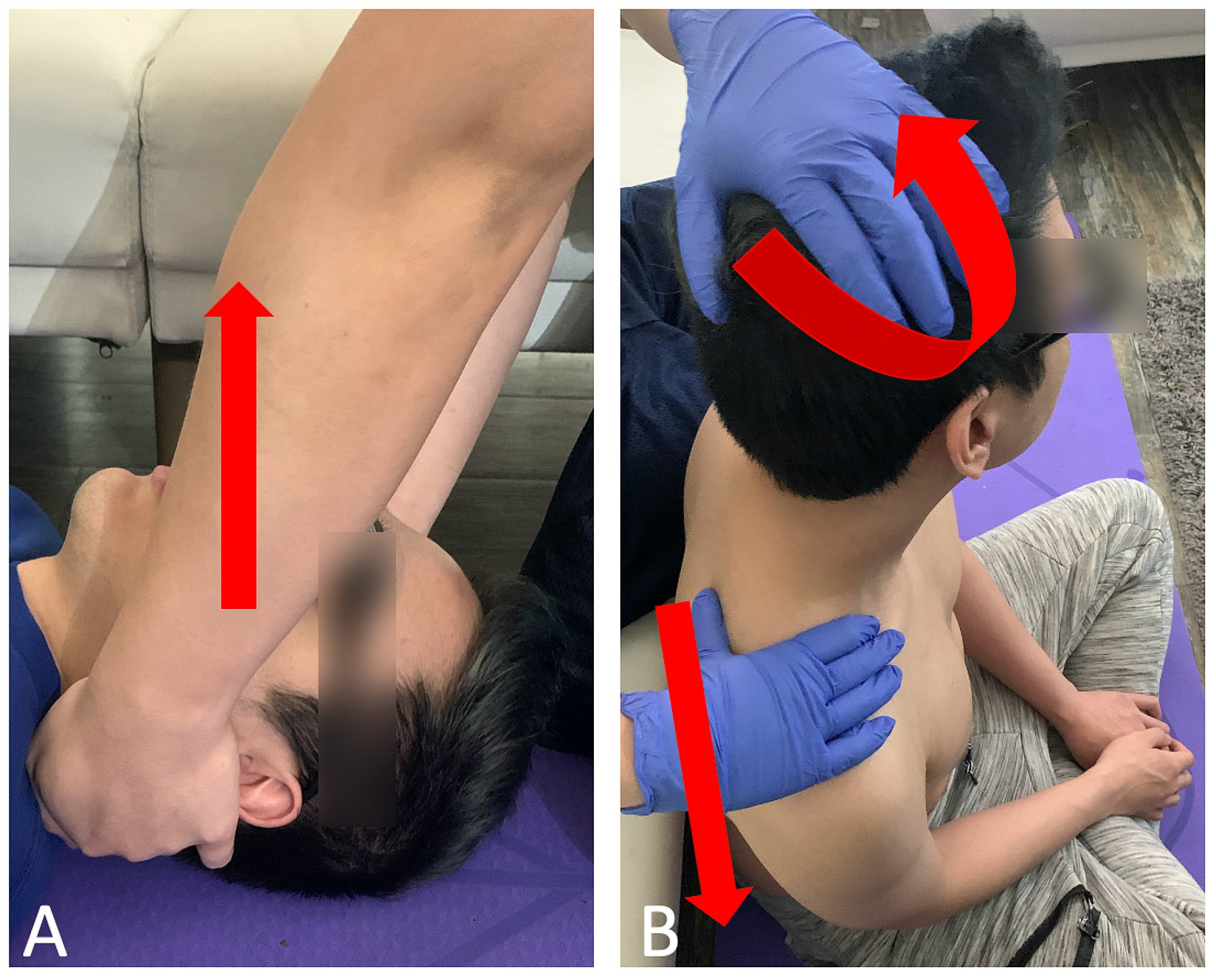
2A reveals common hand placement for myofascial release of the cervical muscles, in which upward tractional force (red arrow) is applied to the cervical paraspinal muscles. 2B shows a variation of muscle energy with hand placement and force vectors (red arrows) for the cervical region.

**Figure 3:**
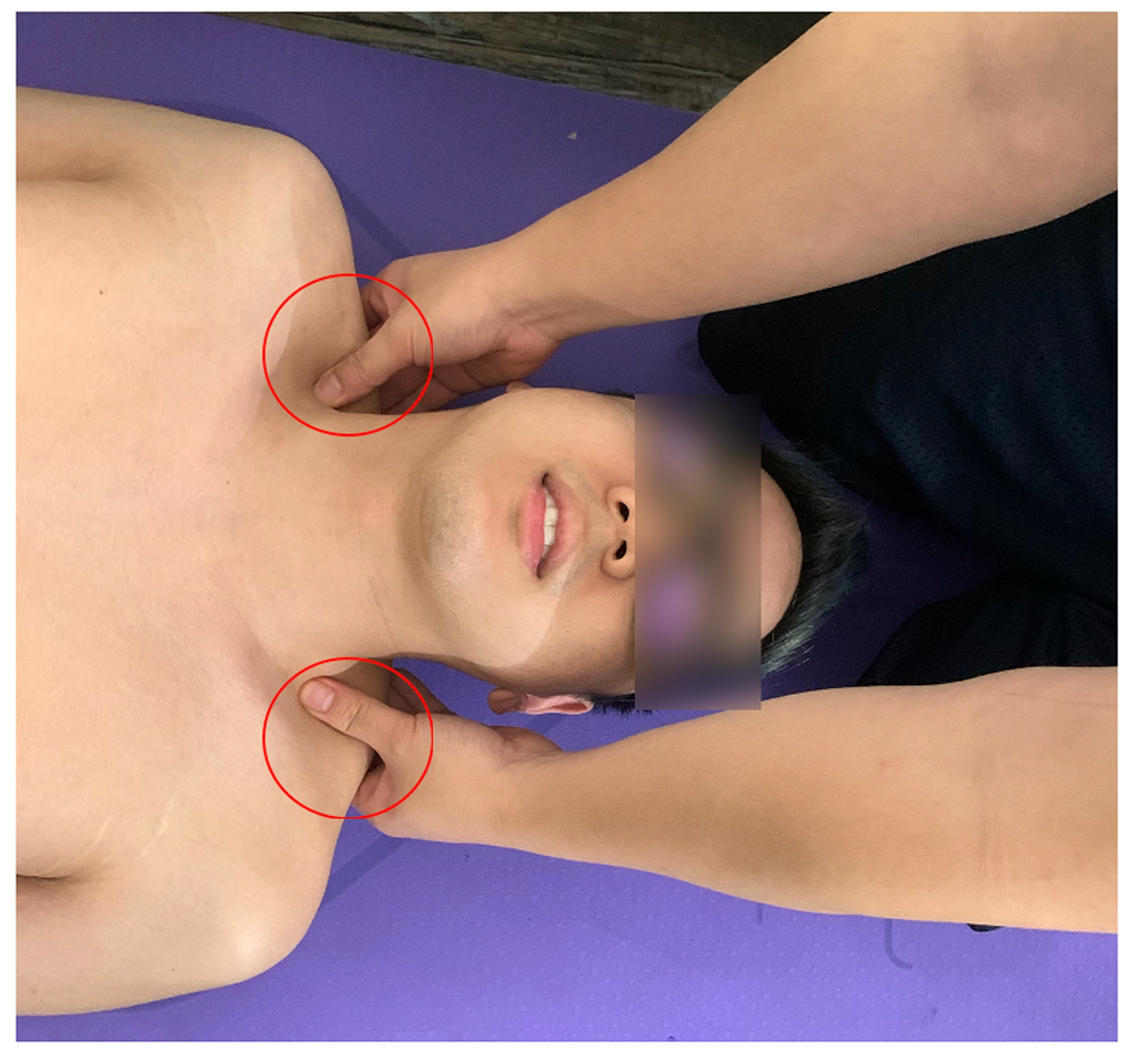
Direct inhibition of the trapezius muscles with hand placement denoted with red circles. This treatment can decrease the tone of hypertonic muscles that contribute to tension headaches.

**Figure 4:**
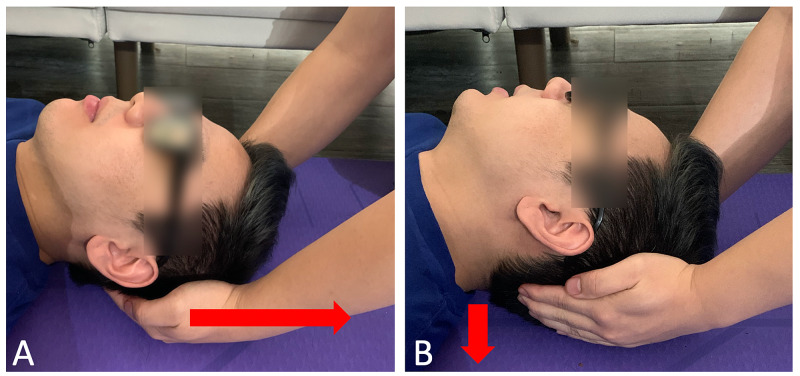
4A: Suboccipital stretch with hand placement at the suboccipital groove and force vectors (red arrow). B. Active resistance and force of the patient engagement cervical muscles toward the ground (red arrow).

The patient consented to osteopathic manipulative treatment and for the inclusion of medical information for publication. OMT for thoracic dysfunctions was addressed with myofascial release and muscle energy techniques (Figure 2). Direct inhibition of the trapezius muscles was applied bilaterally (Figure 3). The treatment sequence was completed with a stretch of the suboccipital muscles with active resistance (Figure 4). The treatment course lasted fifteen minutes after which reassessment revealed resolution of the previously noted somatic dysfunctions. The patient noted subjective improvement in his headache. Upon check out from the clinic, the patient was instructed on various stretching exercises to improve seated posture and decrease muscle strain. Three weeks later, the patient returned for follow-up and reported complete resolution of his symptoms.

## Discussion

Tension-type headaches (TTH) account for 90% of all headaches diagnosed by healthcare professionals [11]. Classically manifesting as a non-pulsatile, bandlike pressure that wraps around the head, patients often self-medicate with non-steroidal anti-inflammatory or analgesic drugs, such as ibuprofen or aspirin [12]. Due to its sensitive nature and relatively quick response to non-prescription based medication, the true incidence and prevalence of TTH are likely higher than what is clinically reported or researched [13]. Recurrence is common as modifiable risk factors associated with TTH are often not addressed, resulting in chronic exacerbation of tension headache triggers. Tension headaches are associated with young age, poor health, sleep disturbances, anxiety, stress, and poor posture [14]. Patients often seek medical care 1) during the first occurrence of TTH, 2) if the headaches become refractory to over-the-counter medication, 3) if episodes of TTH begin to affect daily life, or 4) if additional symptoms develop, such as photophobia or altered sensorium [11]. As seen in the case above, the patient sought medical care after he exhibited his first tension headache, which was likely secondary to stress and poor posture from prolonged computer use.

**Figure 5:**
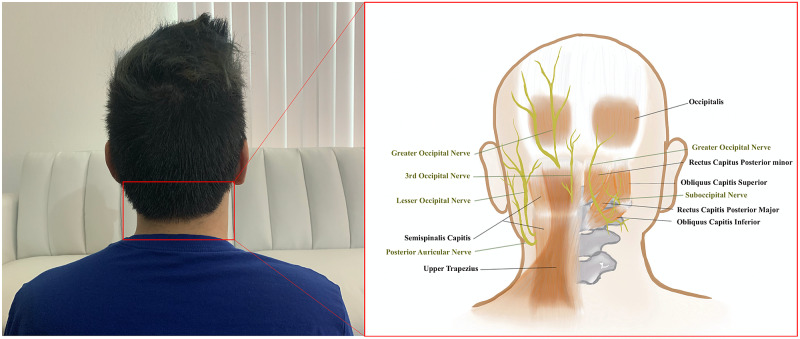
Suboccipital region with illustration inset of associated muscles and nerves in the area. (Original illustration created by YaQun Zhou)

Understanding the underlying mechanisms of TTH is critical in connecting form with function for OMT. The pathologic mechanism for TTH is multifactorial and often includes a combination of personal stress, environmental stimuli, and alteration of central and peripheral pain pathways [15]. The muscles of the suboccipital triangle, rectus capitis posterior major and obliquus capitis superior and inferior, are often implicated as their hypertrophy and asymmetry can result in the compression of the occipital nerve, leading to the typical pain pattern associated with tension headaches (Figure 5). Tensing of the trapezius and erector spinal muscles is a response to psychophysiological stress; this hypertonicity is implicated in TTH, due to the insertion of these muscles near the suboccipital triangle [16]. Fascia also envelops these structures, in which restrictions or abnormalities in the various planes may also similarly result in TTH [17]. Muscle energy and myofascial release are examples of OMT that decrease somatic dysfunction through activation of the Golgi tendon reflex, inhibition of hypertonic muscles, and realignment, which in turn can produce muscle relaxation.

In creating a treatment sequence, osteopathic providers need to not only identify the potential causes of dysfunction but also provide preventive and supportive therapy to mitigate possibilities of recurrence [18,19]. As seen in this case, education and support surrounding stress and posture were given, which ultimately may have contributed to the resolution of symptoms and potentially decreased the risk of future TTH. OMT as TTH prophylaxis has also been investigated, however, due to subjectivity of headache reporting, patient variability in treatment responses, and small sample sizes, it is challenging to devise an optimal OMT strategy in addressing TTH [20]. More significant research, especially with double-blinded studies, is needed further to determine the extent of efficacy in OMT for TTH, however, are difficult due to the difficulty in creating a sham therapy and blinding the operator. Nevertheless, osteopathic providers need to utilize and incorporate OMT into TTH management as it can be a simple, non-invasive option, in addition to standard pain medication and lifestyle changes. For non-osteopathic healthcare providers, a thorough history and physical are crucial in differentiating TTH from other headache etiologies.

## Conclusions

Tension-type headaches are a common complaint seen in primary and urgent care offices. While typically treated with over the counter medications, osteopathic manipulative treatment can be an effective management option to alleviate and prevent future occurrence. Osteopathic and non-osteopathic physicians shoulder consider the use of OMT in TTH, especially when standard pharmaceutical management is inadequate. Addressing the underlying causes of tension-type headaches are also crucial as this can interrupt the initiation of a chronic pain cycle. Further research is needed to understand better and promote the use of osteopathic manipulative medicine in pain complaints such as tension-type headaches.
